# Interlayer charge transfer in supported and suspended MoS_2_/Graphene/MoS_2_ vertical heterostructures

**DOI:** 10.1371/journal.pone.0283834

**Published:** 2023-07-25

**Authors:** Ana K. Rocha Robledo, Mario Flores Salazar, Bárbara A. Muñiz Martínez, Ángel A. Torres-Rosales, Héctor F. Lara-Alfaro, Osvaldo Del Pozo-Zamudio, Edgar A. Cerda-Méndez, Sergio Jiménez-Sandoval, Andres De Luna Bugallo

**Affiliations:** 1 Cinvestav Unidad Querétaro, Querétaro, México; 2 Departamento de Nanotecnología, Centro de Física Aplicada y Tecnología Avanzada, Universidad Nacional Autónoma de México, Querétaro, México; 3 Instituto de Investigación en Comunicación Óptica Universidad Autónoma de San Luis Potosí San Luis Potosí, San Luis, S.L.P. México; Shahrood University of Technology, ISLAMIC REPUBLIC OF IRAN

## Abstract

In this letter, we report on the optical and structural properties of supported and suspended MoS_2_/Graphene/MoS_2_ vertical heterostructures using Raman and photoluminescence (PL) spectroscopies. Vertical heterostructures (VH) are formed by multiple wet transfers on micro-sized holes in SiO_2_/Si substrates, resulting in VH with different configurations. The strong interlayer coupling is confirmed by Raman spectroscopy. Additionally, we observe an enhancement of the PL emission in the three-layer VH (either support or suspended) compared with bare MoS_2_ or MoS_2_/Graphene. This suggests the formation of a spatial type-II band alignment assisted by the graphene layer and thus, the operation of the VH as a n++/metal/n junction.

## 1. Introduction

Semiconductor heterostructure junctions are the basic building block of modern solid-state devices since they enable the engineering of different degrees of freedom such as bandgap, conductivity, and refractive index, among others. Epitaxial growth of thin-film heterostructures is routinely achieved by molecular beam epitaxy (MBE) [1–3] or metal-organic chemical vapor deposition (MOCVD) [4,5]. Even though these techniques offer high-crystallinity and precise control of the thin-film structure during the growth, one of the limiting factors is the choice of the materials since the lattice mismatch has to be near zero to avoid the formation of dislocations [6]. In contrast, due to the absence of dangling bonds and Van der Waals forces between layers [7] the vertical junction of two-dimensional (2D) materials offers an essentially unlimited number of possibilities regarding the choice of the materials and their stacking configuration, including the relative orientation angle between layers which can dramatically change their properties [8,9]. This opens a new gamut of opportunities to explore new physical phenomena and design novel ultra-thin devices.

In particular, different groups have exploited the electronic and optical properties of vertical heterostructures (VH) formed by graphene (Gr) and a single monolayer of molybdenum disulfide in Gr/MoS_2_ [10–14] or Gr/MoS_2_/Gr [15,16] to develop devices. For example, field-effect transistors [3,17] (FET), non-volatile memory cells [18–20] broadband photodetectors [21–25] and chemical sensors [26–31]. Although the properties of Gr/MoS_2_ or Gr/MoS_2_/Gr VHs have been studied, MoS_2_/Gr/MoS_2_ VH remains little explored.

It has been shown by first-principles calculations that the insertion of graphene between two different transition metal dichalcogenides (TMDs) is thermally stable and does not modify their intrinsic band structure. In addition, the resulting heterostructure configuration considerably enhances the interlayer hopping of photogenerated carriers between the conduction (CB) and valence band (VB) of the materials [32]. Experimentally, photoluminescence (PL) and Raman spectroscopy have been demonstrated to be reliable non-invasive techniques to monitor the interlayer charge transfer in VHs since the optical phonons and the radiative and non-radiative recombination paths are highly sensitive to charge doping [33–35].The novelty of our work stems from using the same TMDs (MoS_2_ monolayer) separated by a graphene layer to demonstrate that a similar charge transfer mechanism can also be achieved using the same semiconductor layered materials.

In this work, we study the optical properties of MoS_2_/Gr/MoS_2_ VH supported on SiO_2_ and suspended on micro-sized holes by means of Raman and temperature-dependent micro-photoluminescence (PL) spectroscopies. The technique used to fabricate the VHs results in different stacking configurations (MoS_2_, MoS_2_/Gr, and MoS_2_/Gr/MoS_2_ either supported or suspended) which allows to compare their properties. The position and width of the MoS_2_ and graphene Raman bands reveal strong interlayer coupling between the layers. The PL intensity quenching observed in MoS_2_/Gr VH is attributed to the charge transfer mechanism from MoS_2_ towards graphene. In stark contrast, the PL intensity of the MoS_2_/Gr/MoS_2_ VH, either suspended or supported, is significantly larger than the rest of the above-mentioned configurations, which is indicative of different electronic properties. We propose that the observed PL enhancement is originated from the formation of a spatial type-II band alignment induced by the insertion of the graphene layer between the MoS_2_ monolayers.

## 2. Materials and methods

### 2.1 Sample preparation

Growth of MoS_2_: MoS_2_ crystals were synthesized using an atmospheric pressure chemical vapor deposition (APCVD) system on silicon dioxide (SiO_2_) substrates (S1a). The substrates were immersed and cleaned using an ultrasonic cleaner in acetone and isopropanol baths. The substrate is then placed on top of an alumina boat containing molybdenum dioxide (MoO_2_ Sigma Aldrich 99%) located at the center of the chamber, a second boat containing sulfur (99.5% Alfa Aesar) was loaded 15 cm away from the furnace center. MoS_2_ single layers were principally obtained by raising the temperature furnace at 750 at a 50°C /min ramp using 100 sccm argon flux as a carrier gas and immediately cooling the system after reaching 750°C. Optical and atomic force microscope images confirm the presence of monolayers (S1B and S1C Fig).

Growth of graphene: Large-area graphene layers were grown on 25 μm thick Cu foils (Sigma-Aldrich, 99.98%) as substrate in a low-pressure CVD chamber. The cleaning of the substrates consisted of different baths with ac. nitric acid (5.4% by volume) 30 seconds, acetic acid (4% by volume) 60 seconds, acetone, isopropanol and drying with a flow of nitrogen gas. The Cu foil was annealed at 1010°C for 30 min in 2 sccm H_2_, after this time the H_2_ was turned off and a CH_4_ flow of 20 sccm was introduced into the chamber for 30 min to promote growth, to cool the chamber 50 sccm of Ar.

### 2.2 Microphotoluminescence and Raman characterization

Raman and PL spectra were measured at RT using a micro-Raman spectrometer (LabRAM HR Evolution, HORIBA), and a laser excitation wavelength of 488 nm with a laser power of 0.5 mW was used on the sample, both Raman and PL spectra were measured with a 600 gr/ mm^-1^ grating. The laser radiation was focused onto the substrate surface with a spot of 1 μm diameter using a 100X microscope objective with 0.4 numerical aperture. For measurements at low temperature, the sample was placed in a closed-cycle helium cooled cryostat and examined in micro-PL configuration. A 515 nm diode laser focused with a 50x microscope objective was used to excite PL The emission is analyzed with a spectrometer (HORIBA iHR550) equipped with a CCD camera.

## 3. Results and discussion

Micrometer-sized holes on the SiO_2_/Si substrates were defined using optical lithography. The sample was selectively wet-etched using HF to form holes of approximately 1 μm depth. Subsequently, the MoS_2_/Gr/MoS_2_ heterostructure was fabricated by multiple transferring of CVD MoS_2_ crystals and graphene using the cellulose acetate-assisted method [36]. Details about the growth of MoS_2_ flakes and graphene layers can be found in the methods section. Finally, a thermal annealing treatment at 300°C under argon atmosphere was carried out for two hours to improve the coupling between each material.

**Fig 1:**
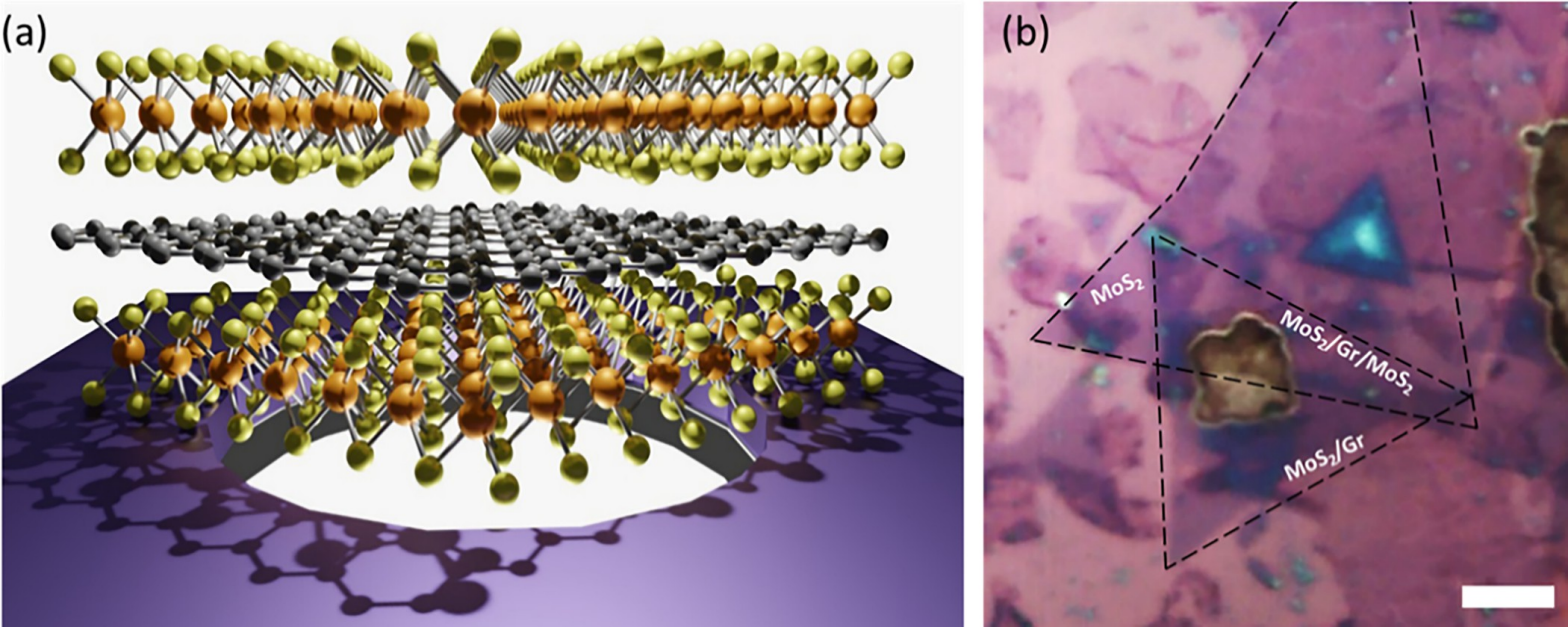
(a) Schematic representation of the MoS_2_/Gr /MoS_2_ vertical heterostructure on SiO_2_ substrates containing micrometer-sized holes. (b) Optical image of the heterostructure (bar scale corresponds to 7 μm).

Fig 1A and 1B show the schematic and optical images of the samples after the artificial heterostructure assembly. The resulting sample consists of different 2D structures, including MoS_2_, MoS_2_/Gr, and MoS_2_/Gr/MoS_2_ either suspended on the hole or supported on the SiO_2_ substrate (A step by step schematic and optical images are shown in S2 and S3 Figs). For simplicity in the following, we will label bilayer and trilayer to make reference to MoS_2_/Gr and MoS_2_/Gr/MoS_2_ VHs repectively.

**Fig 2:**
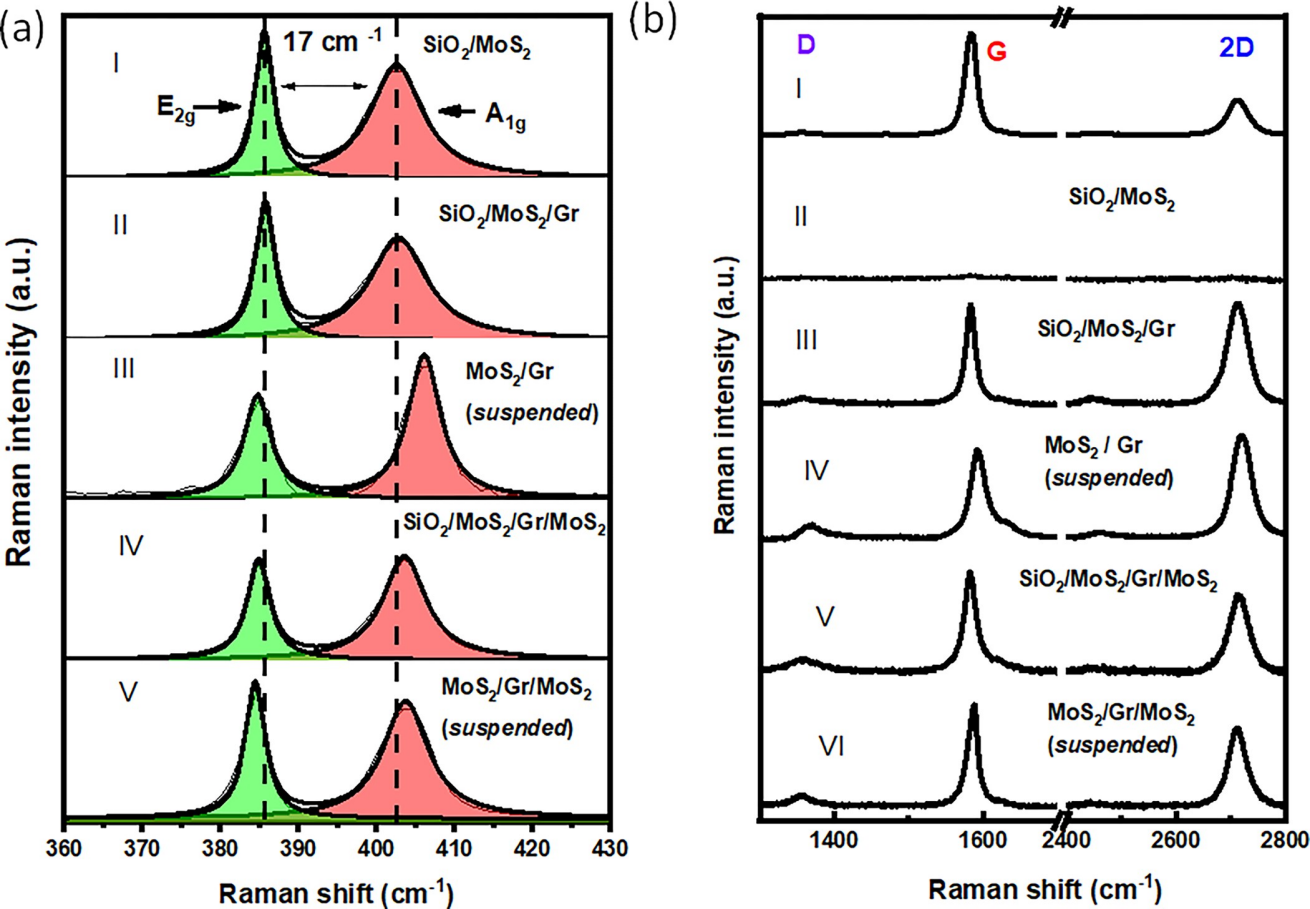
(a) and (b) Raman spectra recorded on different Raman shift range for the different to the vertical heterostructure stacking configurations.

Fig 2A and 2B display the Raman spectra recorded on the different structures laying on SiO_2_ and suspended on the hole. The spatial mapping of the intensity of the Raman signals corresponding to of the A_1g_ and G bands over the whole sample was performed to further demonstrate the coexistence of both materials after the multiple transfer process (S4 Fig). The in-plane E_2g_ and out-of-plane A_1g_ modes of bare MoS_2_ supported on SiO_2_ are centered around 385.7 and 402.6 cm^-1^. The difference wavevector of 17 cm ^1^, confirm the presence of a single MoS_2_ layer [37]. The A_1g_ peak remains around the same position for all the structures except for the suspended bilayer where a strong blue-shift is noticed. The blue-shift can be ascribed to a phonon renormalization due a different carrier concentration compared with other VHs revealing the strong interlayer coupling between the two materials [35]. Interestingly, the position of the E_2g_ and A_1g_ Raman bands measured on the suspended and supported trilayer are very similar to bare MoS_2_, and the difference in wavenumber (18 cm^-1^) indicates that the top and bottom MoS_2_ crystals be considered as monolayers. Finally, considering wavenumber value and the ratio of the G and 2D Raman bands, the Lorentzian fitting of the 2D peak, and the wavelength excitation (488nm), we confirm that the thickness of the trilayer VH is indeed three monolayers (S5 Fig and S1 Table).

Density functional theory calculations [38] and differential reflectance spectroscopy [39] have demonstrated that in a bilayer VH, the A_1g_ blueshift can be attributed to the modification of the charge concentration in the MoS_2_ due to charge transfer from MoS_2_ to the graphene. Note, that this blue-shift is larger in the suspended bilayer than in the supported ones, where charges on the substrate may screen these effects (see curves II and III in Fig 2(A)). Further evidence of the charge transfer in the bilayer from MoS_2_ to Gr is the 9 cm-^1^ blue- shift of the 2D Raman mode of graphene in the VHs (curve IV in Fig 2(B)).

**Fig 3:**
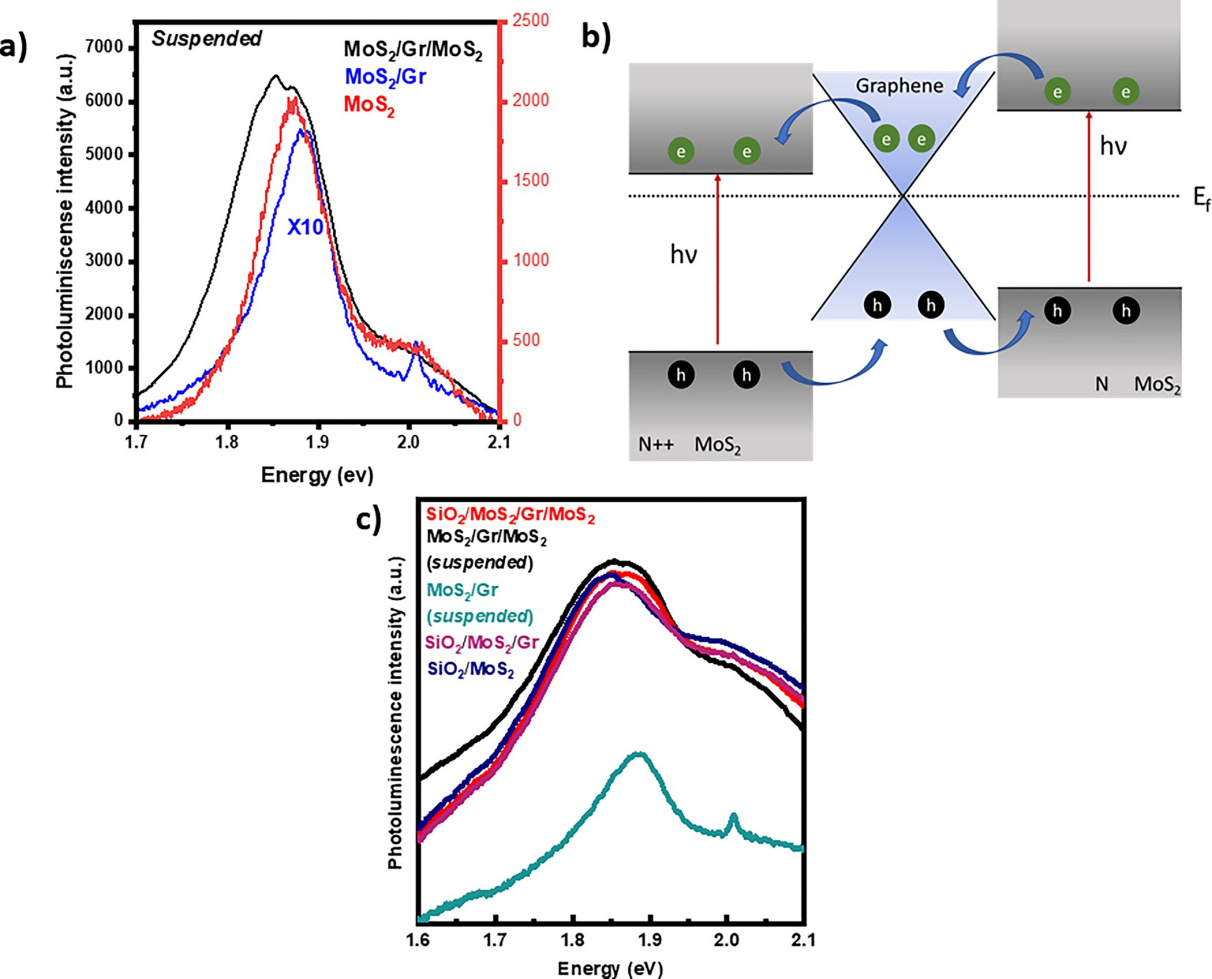
(a) PL emission of suspended MoS_2_, MoS_2_/Gr, and MoS_2_/Gr/MoS_2_, recorded at room temperature (b) Proposed band alignment of MoS_2_/Gr/MoS_2_ heterostructure (c) Comparison of the PL spectra between suspended and supported structures.

Having established the existence of charge transfer from MoS_2_ to Gr, we investigate and compare the PL emission at RT of the three suspended VHs (Fig 3A). For comparison purposes, we measured the PL spectra of the isolated MoS_2_ flake suspended over a hole in a separate sample (S6 Fig). The three samples present two emission features, between 1.85 and 1.88 eV, and another at 2.05 eV, commonly attributed to, respectively, the so-called A and B excitons associated to transitions the direct transition channels [40] from the highest spin-split valence band to the lowest conduction band at the K point of the Brillouin zone. One can make several interesting observations from the PL spectra. First, the signal of the exciton A in the bare MoS_2_ sample peaks around 1.88 eV with a full half-maximum width (FWHM) of 80 meV. In the case of the bilayer, the PL peaks at 1.87 eV and has a similar FWHM than bare MoS_2_. In contrast, there is a notable broadening of the PL spectrum of the trilayer. From the deconvolution of this luminescence spectrum (Figs S7 and 5A), we found a third contribution to the PL signal assigned to the negatively charged exciton or trion X^-^. Note that there is as well significant variation in the PL signal intensity, which will be discussed later.

The emergence of the trion signal in the trilayer indicates a larger carrier concentration in the MoS_2_ than in the other two configurations measured. To explain this feature, we propose an analogous mechanism as that described in Ref 32., where two TMDs monolayers separated by graphene form a spatial type-II band-alignment. In our case, the trilayer forms an n++ semiconductor/graphene/n semiconductor junction, where the photo-excited of the top MoS_2_ monolayer are quickly transferred to the graphene and immediately diffuse towards the conduction band of the bottom MoS_2_ monolayer, promoting the formation of the trions (Fig 3B). Further evidence of this is found in the Raman spectra from the supported and suspended trilayer VHs, where the shift of the 2D band related to doping of graphene is 6 cm^-1^, which was smaller than for the bilayer. The smaller blue-shift indicates a smaller carrier concentration in the graphene, demonstrating that acts as a charge mediator between both MoS_2_ layers.

We now turn our attention to the PL intensity emission recorded VH configurations (Fig 3C). We observe that while the signal intensity of the bilayer supported on SiO_2_ decreases in comparison with bare MoS_2_ on SiO_2_, it dramatically diminishes for the suspended bilayer heterostructure. As pointed out in the discussion about Raman characterization, the PL quenching is attributed to the interlayer charge transfer that modifies the recombination paths of the photogenerated species A and B excitons and the charged exciton or trion [38]. In the case of the suspended bilayer, the PL intensity further decreases by the reduction of amplification by optical interference due to the lack of the SiO_2_ spacer [41]. The effect of the substrate can also be observed in S8 Fig. Since the recombination processes in PL are sensitive to laser power [42], the change in linewidths can be related to the relative intensities of the exciton A and the trion on the supported MoS_2_ (S8A Fig) under different excitation conditions. It can be noticed that the PL spectra of the bilayer deposited directly on the SiO_2_ substrate broadens as the laser power increases (S8B Fig) while that of the suspended bilayer remains unchanged (S8C Fig), indicating that the trion formation in the former takes place between excitons and free charges present on the SiO_2_ substrate. The substrate effect can also be observed in the case of the supported trilayer (S8D Fig). In contrast to the suspended bilayer, the suspended trilayer VH does show an important broadening, further supporting our assertions regarding that the formation of trions in this VH is related to graphene rather than to the free charges on SiO_2_.

Moreover, the PL measured on the trilayer displays a similar or even slightly larger intensity (Fig 3C) than bare MoS_2_ regardless of the VH being suspended or supported on SiO_2_. This can be ascribed to the internal loop created by the formation of the spatial type II band alignment, which would lead high emission efficiency due to the short times of the e-h recombination process in the stacking trilayer, explaining the high PL emission on the supported or suspended regions.

**Fig 4:**
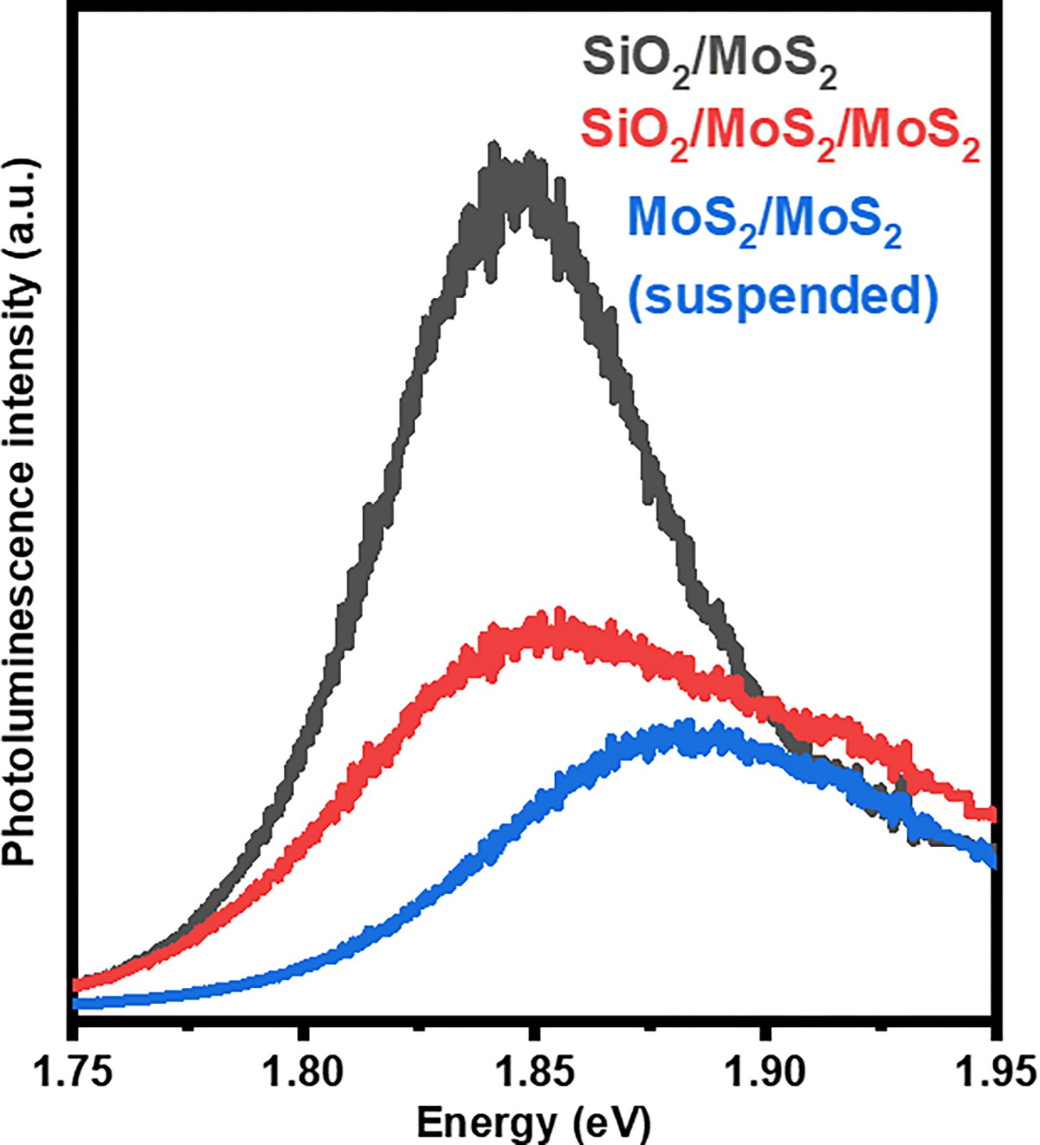
Photoluminescence spectra comparison between bare MoS_2_ and MoS_2_/MoS_2_ suspended and supported VHs.

To further investigate the role of the graphene layer in the trilayer, we measured the PL of MoS_2_ bilayers under the same conditions as the trilayer VH. Fig 4 shows that the PL emission of the bilayer presents a significant quenching compared to bare monolayer MoS_2_ on SiO_2_ either when suspended or supported on SiO_2_. This is well-known to be related to the emergence of the indirect bandgap recombination path [43], showing the importance of the graphene layer to form the spatial type II alignment while preserving the direct character of the transition. Interestingly, this point towards the possibility to use the graphene layer to control the charge density on the MoS_2_ layers by applying an external gate bias to control the Fermi energy level.

**Fig 5:**
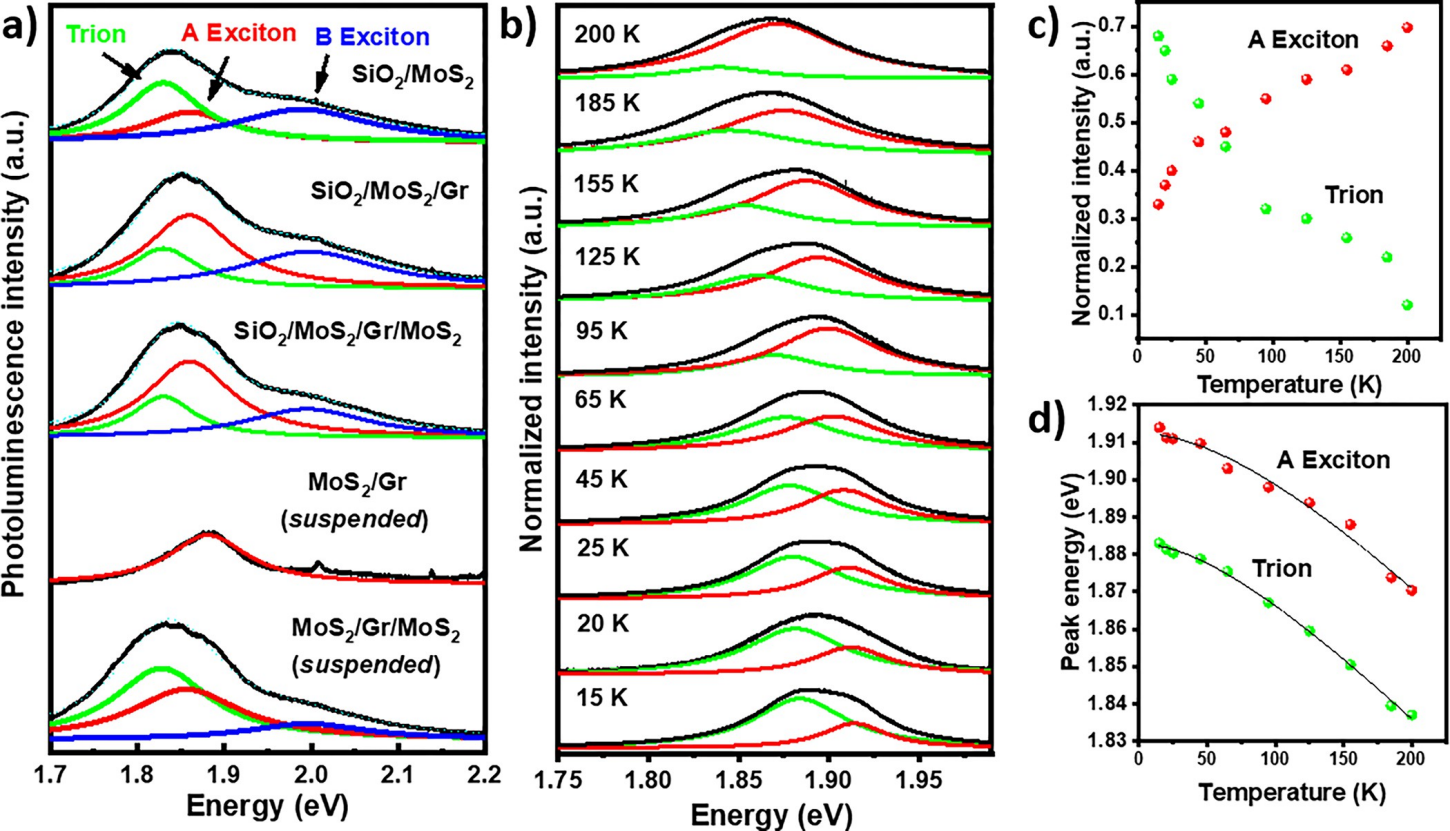
(a) Lorentzian fitting of the PL spectra collected from the different VH configurations of the sample. (b) Collected PL spectra of the suspended MoS_2_/Gr/MoS_2_ VH at different temperatures. (c- d) Intensity and peak position of exciton A and trion (X^-^) as a function of the temperature.

As mentioned before, the PL spectra of the trilayer presents broadening. To identify and compare the different PL features in all the samples, we performed a Lorentzian fitting of the PL spectra, revealing three excitonic resonances peaked around 2.05, 1.87, and 1.83 eV (Fig 5A). In one hand, while there is significant emission due to the trion formation in bare MoS_2_ on SiO_2_, when graphene is stacked on MoS_2_, exciton A becomes the predominant contribution (top curves of Figs 5A and S9A). On the other hand, the integrated intensity area of the trion feature (S9B Fig) increases with the excitation intensity only for the suspended trilayer, suggesting that part of the electrons recombine at the surface of the substrate when the trilayer is laying directly on the SiO_2,_ limiting the trion recombination path.

Finally, we performed micro-PL in the temperature range from 15K-200K on the trilayer suspended region. It is noteworthy that no PL signals related to bound excitons were observed, indicating low defect densities in both MoS_2_ CVD monolayers forming the VH. The luminescence of both excitonic resonances, A exciton and trion, progressively blueshift with decreasing the temperature from 200K to 15K. By fitting the spectra, we noted that the normalized intensity of the exciton A decreases while the trion intensity increases at lower temperatures (Fig 5C). In addition, it is possible to reproduce the variation of the peak position of each PL feature applying the Varshni expression [44]:

$$E(T)=E(0)-\frac{\alpha T^{2}}{T-\beta }$$ (1)

where E (0) is the energy peak position of the trion or exciton A at 0K and α and β the fitting parameters of the material. Using the data shown in Fig 5D we found the following values: for the exciton A α = 0.33meV/K and β = 159K and for the trion α = 0.37 meV/K and β = 143K. These results along with the obtained values for the fitting parameters of the Varshni equation, are in good agreement with low temperature PL experiments performed on single MoS_2_ [45,46] indicating that the recombination process of the trilayer is close to an individual MoS_2_ layer.

## 4. Conclusions

In summary, we have studied the optical properties of trilayer (MoS_2_/Gr/MoS_2_) heterostructures suspended and supported on SiO_2_ substrates. The photoluminescence emission intensity from the trilayer structures indicates the formation of an n++ semiconductor/graphene/n semiconductor, resulting in a spatial type II band alignment where graphene acts as a charge mediator between the MoS_2_ monolayers. The importance of our results derives from the fact that a graphene layer laying between two monolayers of the same material forms a type II band alignment, which is normally achieved using different TMDs. Moreover, the graphene layer can be electrically back-gated to move upward or downwards the Fermi level and control the carrier density in the MoS_2_ monolayers, either to enhance or decrease the PL emission. Therefore, we propose that this configuration could be used as an electrical tunable interface to develop novel optoelectronic and electronic devices.

## Supporting information

S1 Fig MoS_2_ CVD growth and morphology characterization. (a) APCVD set up view used to the MoS_2_ growth deposited on Si/SiO_2_ substrates. (b) Optical and (c) atomic force microscopy images of MoS_2_ crystals. All bar scales correspond to 10 μm.
(TIF)

S2 Fig Schematic representation of transfer process of MoS_2_-Gr system. (a) Optical image of MoS_2_-Gr system b-d) Schematic representation of transfer process.
(TIF)

S3 Fig Schematic representation of transfer process of MoS_2_-Gr-MoS_2_ system a) Optical image of MoS_2_-Gr-MoS_2_ system b-d) schematic representation of transfer process.
(TIF)

S4 Fig Raman intensity mapping of the A_1g_ and G bands in the study region. (TIF)

S5 Fig Lorentzian Raman deconvolution of MoS_2_-Gr-MoS_2_ system. (TIF)

S6 Fig 6Optical image of MoS_2_ monolayer on SiO_2_ substrate with a micrometer-sized hole. (TIF)

S7 Fig Lorentzian deconvolution of PL spectra of MoS_2_ suspended. (TIF)

S8 Fig Laser power dependence photoluminescence spectrums (a) SiO_2_/MoS_2_ and (b) SiO_2_/MoS_2_/Gr (c) MoS_2_/Gr (d) SiO_2_/MoS_2_/Gr/MoS_2_ and (e) MoS_2_/Gr/MoS_2_.
(TIF)

S9 Fig A exciton and trion area (Fig 5A). (TIF)

S1 Table I(G)/2(G) ratio for MoS_2_/Gr/MoS_2_ system (488 nm). The ratio of around 1.14 confirms the presence of Gr trilayer.
(TIF)

## References

[1] Chaves A., Azadani J.G., Alsalman H., da Costa D.R., Frisenda R., Chaves A.J., et al, Bandgap engineering of two-dimensional semiconductor materials, Npj 2D Mater. Appl. 4 (2020). 10.1038/s41699-020-00162-4.

[2] Yuan X., Tang L., Liu S., Wang P., Chen Z., Zhang C., et al, Arrayed van der waals vertical heterostructures based on 2d GaSe grown by molecular beam epitaxy, Nano Lett. 15 (2015) 3571–3577. doi: 10.1021/acs.nanolett.5b01058 25923041

[3] Nakano M., Iwasa Y., Emergent transport phenomena in MBE-grown 2D materials and their heterostructures, in: 2019 Compd. Semicond. Week, 2019: p. 1. 10.1109/ICIPRM.2019.8819228.

[4] Lee D.H., Sim Y., Wang J., Kwon S.Y., Metal-organic chemical vapor deposition of 2D van der Waals materials—The challenges and the extensive future opportunities, APL Mater. 8 (2020). 10.1063/1.5142601.

[5] Lin Y.-C., Ghosh R.K., Addou R., Lu N., Eichfeld S.M., Zhu H., et al, Atomically thin resonant tunnel diodes built from synthetic van der Waals heterostructures., Nat. Commun. 6 (2015) 7311. doi: 10.1038/ncomms8311 26088295 PMC4557306

[6] Liu X., Balla I., Bergeron H., Campbell G.P., Bedzyk M.J., Hersam M.C., Rotationally Commensurate Growth of MoS_2_ on Epitaxial Graphene, ACS Nano. 10 (2016) 1067–1075. doi: 10.1021/acsnano.5b06398 26565112

[7] Bierwagen O., White M.E., Tsai M.-Y., Speck J.S., Chapter 15—MBE of transparent semiconducting oxides, in: Henini M.B.T.-M.B.E. (Ed.), Elsevier, Oxford, 2013: pp. 347–367. 10.1016/B978-0-12-387839-7.00015-4.

[8] Liu Y., Weiss N.O., Duan X., Cheng H.C., Huang Y., Duan X., Van der Waals heterostructures and devices, Nat. Rev. Mater. 1 (2016). 10.1038/natrevmats.2016.42.

[9] Zhu S., Pochet P., Johnson H.T., Controlling Rotation of Two-Dimensional Material Flakes, ACS Nano. 13 (2019) 6925–6931. doi: 10.1021/acsnano.9b01794 31082256

[10] Muñoz R., López-Elvira E., Munuera C., Frisenda R., Sánchez-Sánchez C., Martín-Gago J.Á., et al, Direct growth of graphene-MoS_2_ heterostructure: Tailored interface for advanced devices, Appl. Surf. Sci. 581 (2022). 10.1016/j.apsusc.2021.151858.

[11] Jin Y., Joo M.K., Moon B.H., Kim H., Lee S., Jeong H.Y., et al, Coulomb drag transistor using a graphene and MoS_2_ heterostructure, Commun. Phys. 3 (2020) 1–8. 10.1038/s42005-020-00461-8.

[12] Xu H., He D., Fu M., Wang W., Wu H., Wang Y., Optical identification of MoS_2/graphene heterostructure on SiO_2_/Si substrate, Opt. Express. 22 (2014) 15969. 10.1364/oe.22.015969. 24977852

[13] Kim T., Fan S., Lee S., Joo M.K., Lee Y.H., High-mobility junction field-effect transistor via graphene/MoS_2_ heterointerface, Sci. Rep. 10 (2020) 1–8. 10.1038/s41598-020-70038-6. 32753604 PMC7403303

[14] Wang Z., Chen Q., Wang J., Electronic structure of twisted bilayers of graphene/MoS_2_ and MoS2/MoS2, J. Phys. Chem. C. 119 (2015) 4752–4758. 10.1021/jp507751p.

[15] Kumar S., Singh A., Nivedan A., Kumar S., Yun S. J., Lee, et al. Sub‐bandgap activated charges transfer in a graphene‐MoS_2_‐graphene heterostructure. Nano Select (2021), 2(10), 2019–2028. 10.1002/nano.202000159.

[16] Elder R.M., Neupane M.R., Chantawansri T.L., Stacking order dependent mechanical properties of graphene/MoS_2_ bilayer and trilayer heterostructures, Appl. Phys. Lett. 107 (2015). 10.1063/1.4928752.

[17] Farooq Khan M., Arslan Shehzad M., Zahir Iqbal M., Waqas Iqbal M., Nazir G., Seo Y., et al A facile route to a high-quality graphene/MoS_2_ vertical field-effect transistor with gate-modulated photocurrent response, J. Mater. Chem. C. 5 (2017) 2337–2343. 10.1039/C6TC04716E.

[18] Roy K., Padmanabhan M., Goswami S., Sai T.P., Ramalingam G., Raghavan S., et al, Graphene-MoS_2_ hybrid structures for multifunctional photoresponsive memory devices, Nat. Nanotechnol. 8 (2013) 826–830. 10.1038/nnano.2013.206. 24141541

[19] Bertolazzi S., Krasnozhon D., Kis A., Nonvolatile Memory Cells Based on MoS2/Graphene Heterostructures, ACS Nano. 7 (2013) 3246–3252. doi: 10.1021/nn3059136 23510133

[20] Wang X., Xie W., Bin Xu J., Graphene based non-volatile memory devices, Adv. Mater. 26 (2014) 5496–5503. doi: 10.1002/adma.201306041 24497002

[21] Liu B., Chen Y., You C., Liu Y., Kong X., Li J., et al, High performance photodetector based on graphene/MoS_2_/graphene lateral heterostrurcture with Schottky junctions, J. Alloys Compd. 779 (2019) 140–146. 10.1016/j.jallcom.2018.11.165.

[22] Thai K.Y., Park I., Kim B.J., Hoang A.T., Na Y., Park C.U., et al, MoS_2_/Graphene Photodetector Array with Strain-Modulated Photoresponse up to the Near-Infrared Regime, ACS Nano. 15 (2021) 12836–12846. 10.1021/acsnano.1c04678. 34291913

[23] Zhang T., Wu Z., Chen Y., Zhang Y., Shao Z., Liu L., et al, Graphene/MoS_2_ heterostructure photodetector integrated with silicon nitride micro-ring resonators at visible wavelengths, in: Commun Asia. Photonics Conf., Optica Publishing Group, 2017: p. Su3E.5. 10.1364/ACPC.2017.Su3E.5.

[24] Han P., Marie L. S., Wang Q. X., Quirk N., El Fatimy A., Ishigami M., et al. Highly sensitive MoS_2_ photodetectors with graphene contacts. *Nanotechnology* (2018), 29(20), 20LT01. 10.1088/1361-6528/aab4bb. 29512512

[25] Zhang W., Chuu C.P., Huang J.K., Chen C.H., Tsai M.L., Chang Y.H., et al, Ultrahigh-Gain Photodetectors Based on Atomically Thin Graphene-MoS_2_ Heterostructures, Sci. Rep. 4 (2015) 1–8. 10.1038/srep03826. PMC3899643 24451916

[26] Lin D., Su Z., Wei G., Three-dimensional porous reduced graphene oxide decorated with MoS2 quantum dots for electrochemical determination of hydrogen peroxide, Mater. Today Chem. 7 (2018) 76–83. 10.1016/j.mtchem.2018.02.001.

[27] Jeong J.-M., Yang M., Kim D.S., Lee T.J., Choi B.G., Kim D.H., High performance electrochemical glucose sensor based on three-dimensional MoS_2_/graphene aerogel, J. Colloid Interface Sci. 506 (2017) 379–385. 10.1016/j.jcis.2017.07.061. 28750240

[28] Yoon J., Shin J.-W., Lim J., Mohammadniaei M., Bharate Bapurao G., Lee T., et al, Electrochemical nitric oxide biosensor based on amine-modified MoS_2_/graphene oxide/myoglobin hybrid, Colloids Surfaces B Biointerfaces. 159 (2017) 729–736. 10.1016/j.colsurfb.2017.08.033. 28886511

[29] Yoon J., Lee T., Bapurao G B., Jo J., Oh B.-K., Choi J.-W., Electrochemical H_2_O_2_ biosensor composed of myoglobin on MoS_2_ nanoparticle-graphene oxide hybrid structure, Biosens. Bioelectron. 93 (2017) 14–20. 10.1016/j.bios.2016.11.064. 27955988

[30] Cho B., Yoon J., Lim S.K., Kim A.R., Kim D.-H., Park S.-G., et al, Chemical Sensing of 2D Graphene/MoS_2_ Heterostructure device, ACS Appl. Mater. Interfaces. 7 (2015) 16775–16780. 10.1021/acsami.5b04541. 26161691

[31] Choi J.W., Yoon J., Lim J., Shin M., Lee S.N., Graphene/MoS_2_ nanohybrid for biosensors, Materials (Basel). 14 (2021) 1–22. 10.3390/ma14030518. PMC7865552 33494525

[32] Xia C., Xiong W., Xiao W., Du J., Fang L., Li J., Jia Y., Enhanced Carrier Concentration and Electronic Transport by Inserting Graphene into van der Waals Heterostructures of Transition-Metal Dichalcogenides, Phys. Rev. Appl. 10 (2018) 1. 10.1103/PhysRevApplied.10.024028.

[33] Carozo V., Fujisawa K., Rao R., Kahn E., Cunha J.R., Zhang T., et al, Excitonic processes in atomically-thin MoSe_2_/MoS_2_ vertical heterostructures, 2D Mater. 5 (2018). 10.1088/2053-1583/aacbe8.

[34] Lee T., Choi J.-H., Ahn J.-H., Yoon Y.-G., Rho H., Unveiling the origin of two distinct routes of interlayer charge transfer doping in Bi_2_Te_3_/MoS_2_/SiO_2_ heterostructure, Appl.Surf. Sci. 579 (2022) 152208. 10.1016/j.apsusc.2021.1 52208.

[35] Yang M., Wang L., Hu G., Chen X., Gong P. L., Cong X., et al. (2021). Optical identification of interlayer coupling of graphene/MoS_2_ van der Waals heterostructures. Nano Research, 14, 2241–2246.

[36] Zhang T., Fujisawa K., Granzier-Nakajima T., Zhang F., Lin Z., Kahn E., et al, Clean Transfer of 2D Transition Metal Dichalcogenides Using Cellulose Acetate for Atomic Resolution Characterizations, ACS Appl. Nano Mater. 2 (2019) 5320–5328. 10.1021/acsanm.9b01257.

[37] Jin K., Liu D., Tian Y., Enhancing the interlayer adhesive force in twisted multilayer MoS2 by thermal annealing treatment, Nanotechnology. 26 (2015) 405708. 10.1088/0957-4484/26/40/405708. 26376935

[38] Garcia-Basabe Y., Rocha A.R., Vicentin F.C., Villegas C.E.P., Nascimento R., Romani E.C., et al, Ultrafast charge transfer dynamics pathways in two-dimensional MoS_2_-graphene heterostructures: A core-hole clock approach, Phys. Chem. Chem. Phys. 19 (2017) 29954–29962. 10.1039/c7cp06283d. 29090284

[39] Xu Z., Liu Z., Zhang D., Zhong Z., Norris T.B., Ultrafast dynamics of charge transfer in CVD grown MoS_2_-graphene heterostructure, Appl. Phys. Lett. 119 (2021). 10.1063/5.0060256.

[40] Christopher J.W., Goldberg B.B., Swan A.K., Long tailed trions in monolayer MoS_2_: Temperature dependent asymmetry and resulting red-shift of trion photoluminescence spectra, Sci. Rep. 7 (2017) 1–8. 10.1038/s41598-017-14378-w. 29070869 PMC5656673

[41] Buscema M., Steele G.A., van der Zant H.S.J., Castellanos-Gomez A., The effect of the substrate on the Raman and photoluminescence emission of single-layer MoS_2_, Nano Res. 7 (2014) 561–571. 10.1007/s12274-014-0424-0.

[42] Kaplan D., Gong Y., Mills K., Swaminathan V., Ajayan P.M., Shirodkar S., et al, Excitation intensity dependence of photoluminescence from monolayers of MoS_2_ and WS_2_/MoS_2_ heterostructures, 2D Mater. 3 (2016) 0–27. 10.1088/2053-1583/3/1/015005.

[43] Golovynskyi S., Irfan I., Bosi M., Seravalli L., Datsenko O.I., Golovynska I., et al, Exciton and trion in few-layer MoS_2_: Thickness- and temperature-dependent photoluminescence, Appl. Surf. Sci. 515 (2020) 146033. 10.1016/j.apsusc.2020.146033.

[44] Varshni Y.P., Temperature dependence of the energy gap in semiconductors, Physica. 34 (1967) 149–154. 10.1016/0031-8914(67)90062-6.

[45] Sigiro M., Huang Y.S., Ho C.H., Lin Y.C., Suenaga K., Influence of rhenium on the structural and optical properties of molybdenum disulfide, Jpn. J. Appl. Phys. 54 (2015). 10.7567/JJAP.54.04DH05.

[46] Ko T.S., Huang C.C., Lin D.Y., Optical and transport properties of Ni-MoS_2_, Appl. Sci. 6 (2016) 227. 10.3390/app6080227.

