# Supplementary figures and images for: Interlayer charge transfer in supported and suspended MoS2/Graphene/MoS2 vertical heterostructures
Source: PLoS One. 2023 Jul 25;18(7):e0283834. doi: 10.1371/journal.pone.0283834 (PMC10368229; doi:10.1371/journal.pone.0283834)

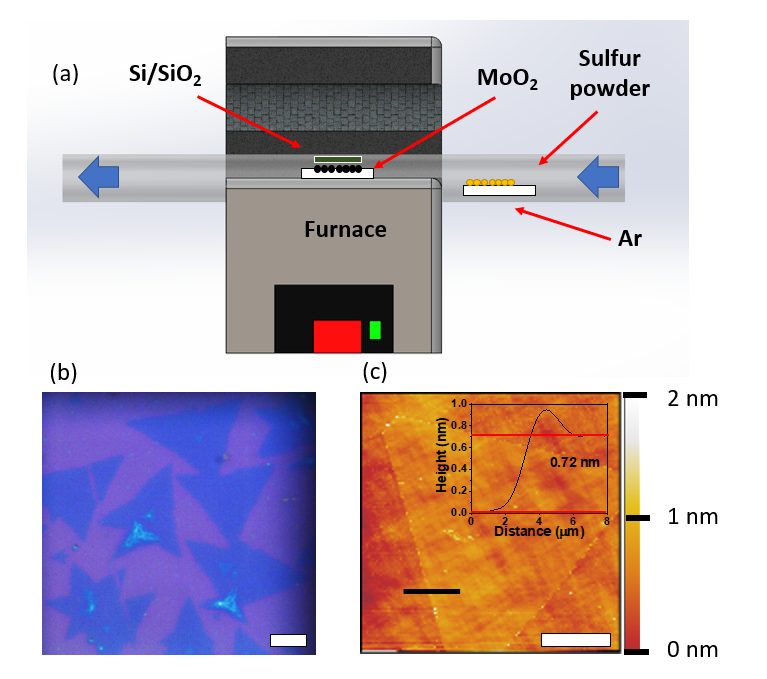

Supplement: S1 Fig — (a) APCVD set up view used to the MoS2 growth deposited on Si/SiO2 substrates. (b) Optical and (c) atomic force microscopy images of MoS2 crystals. All bar scales correspond to 10 μm. (TIF) [file pone.0283834.s001.tif]

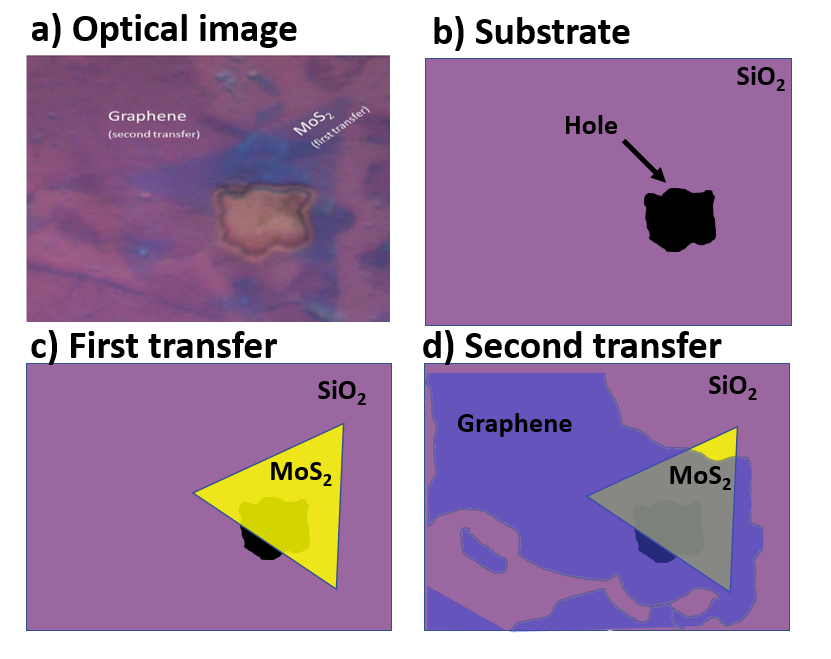

Supplement: S2 Fig — (a) Optical image of MoS2-Gr system b-d) Schematic representation of transfer process. (TIF) [file pone.0283834.s002.tif]

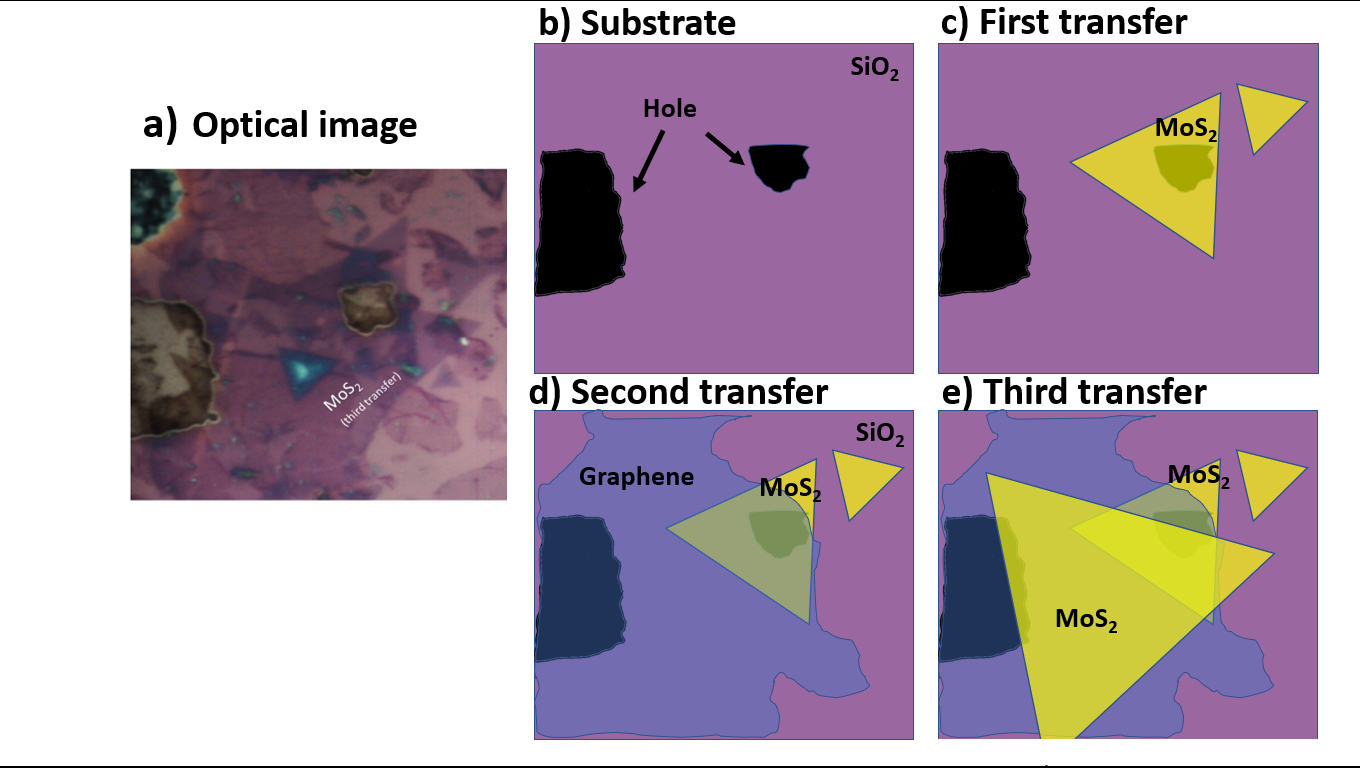

Supplement: S3 Fig — Schematic representation of transfer process of MoS2-Gr-MoS2 system a) Optical image of MoS2-Gr-MoS2 system b-d) schematic representation of transfer process. (TIF) [file pone.0283834.s003.tif]

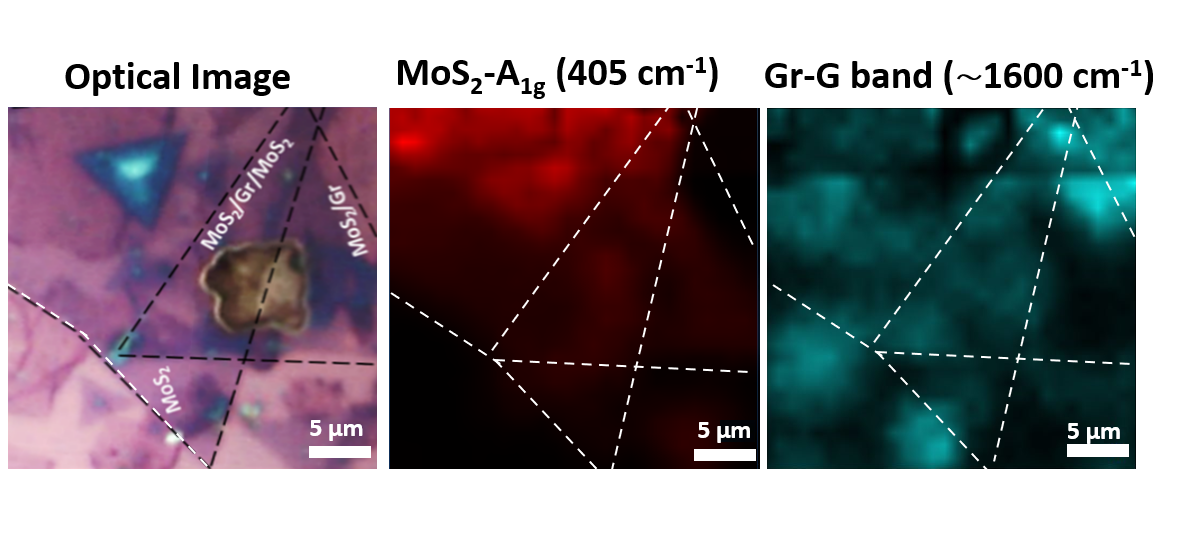

Supplement: S4 Fig — (TIF) [file pone.0283834.s004.tif]

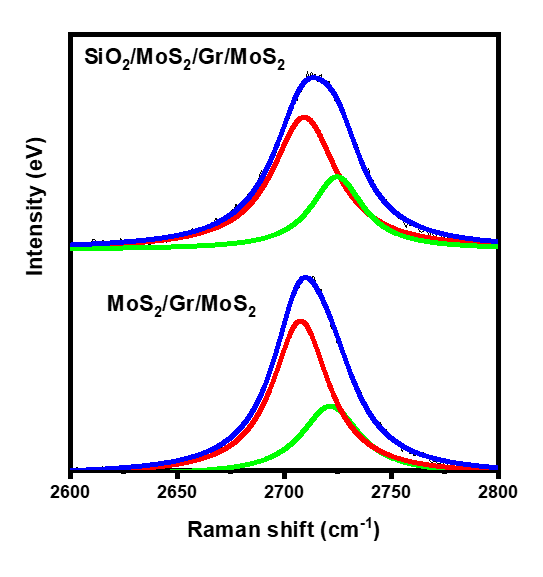

Supplement: S5 Fig — (TIF) [file pone.0283834.s005.tif]

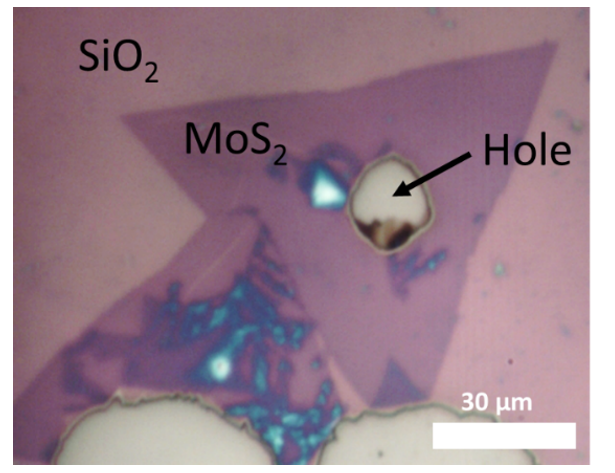

Supplement: S6 Fig — (TIF) [file pone.0283834.s006.tif]

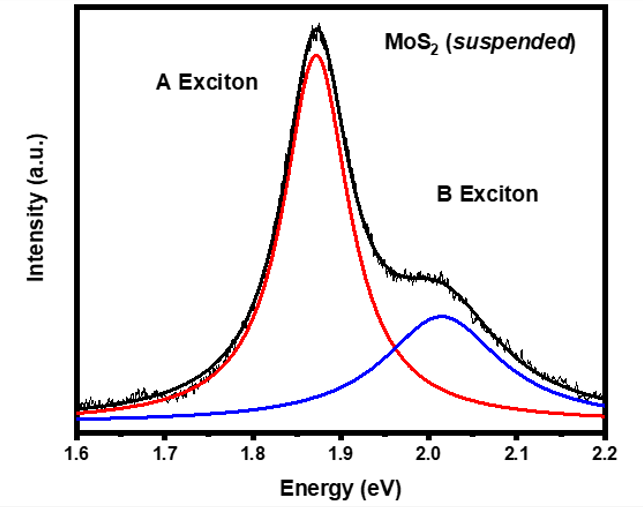

Supplement: S7 Fig — (TIF) [file pone.0283834.s007.tif]

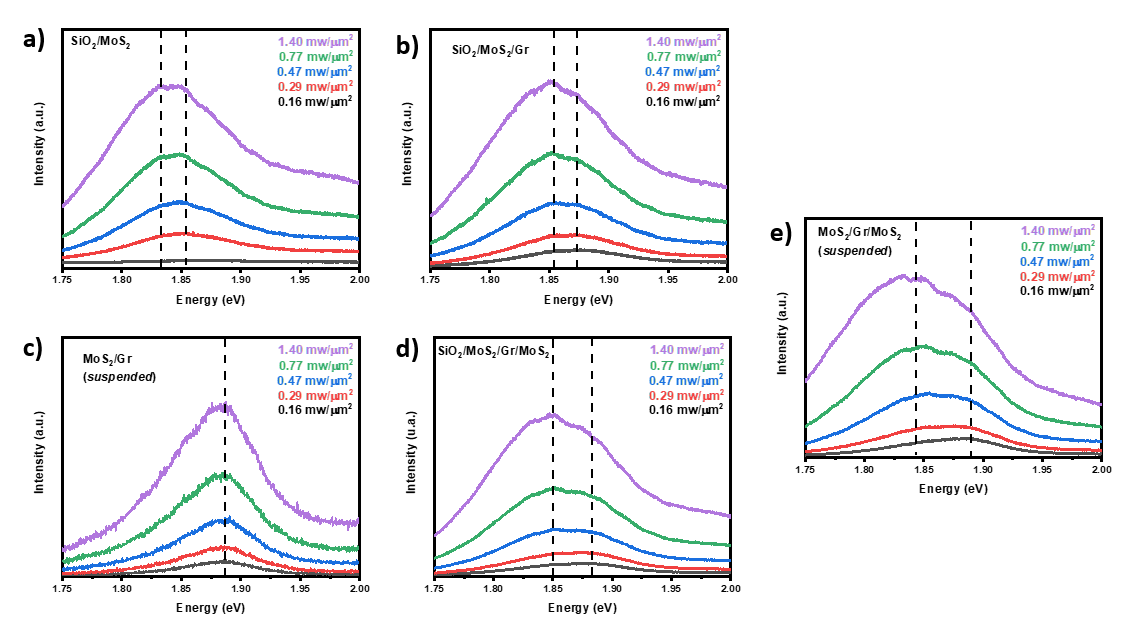

Supplement: S8 Fig — Laser power dependence photoluminescence spectrums (a) SiO2/MoS2 and (b) SiO2/MoS2/Gr (c) MoS2/Gr (d) SiO2/MoS2/Gr/MoS2 and (e) MoS2/Gr/MoS2. (TIF) [file pone.0283834.s008.tif]

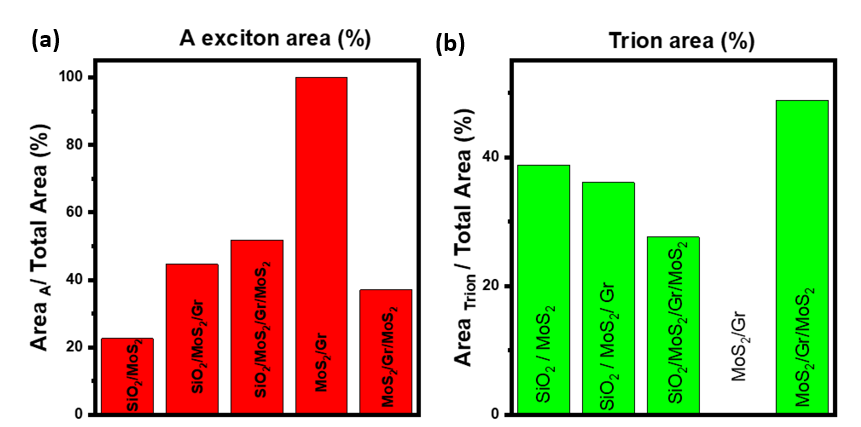

Supplement: S9 Fig — (TIF) [file pone.0283834.s009.tif]

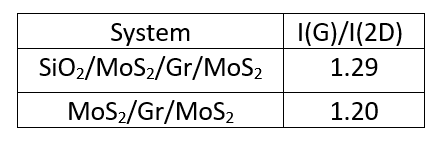

Supplement: S1 Table — The ratio of around 1.14 confirms the presence of Gr trilayer. (TIF) [file pone.0283834.s010.tif]
